# An effective screening model for subjective cognitive decline in community-dwelling older adults based on gait analysis and eye tracking

**DOI:** 10.3389/fnagi.2024.1444375

**Published:** 2024-09-25

**Authors:** Chenxi Hao, Xiaonan Zhang, Junpin An, Wenjing Bao, Fan Yang, Jinyu Chen, Sijia Hou, Zhigang Wang, Shuning Du, Yarong Zhao, Qiuyan Wang, Guowen Min, Yang Li

**Affiliations:** ^1^Department of Neurology, First Hospital of Shanxi Medical University, Taiyuan, China; ^2^Department of Radiology, First Hospital of Shanxi Medical University, Taiyuan, China; ^3^Department of Medical Imaging, Shanxi Medical University, Taiyuan, China; ^4^State Key Laboratory of Computer Science and Beijing Key Lab of Human-Computer Interaction, Institute of Software, Chinese Academy of Sciences, Beijing, China; ^5^Department of First Clinical Medicine, Shanxi Medical University, Taiyuan, China

**Keywords:** subjective cognitive decline, machine learning, gait analysis, eye tracking, screening model, in the community

## Abstract

**Objective:**

To evaluate the effectiveness of multimodal features based on gait analysis and eye tracking for elderly people screening with subjective cognitive decline in the community.

**Methods:**

In the study, 412 cognitively normal older adults aged over 65 years were included. Among them, 230 individuals were diagnosed with non-subjective cognitive decline and 182 with subjective cognitive decline. All participants underwent assessments using three screening tools: the traditional SCD9 scale, gait analysis, and eye tracking. The gait analysis involved three tasks: the single task, the counting backwards dual task, and the naming animals dual task. Eye tracking included six paradigms: smooth pursuit, median fixation, lateral fixation, overlap saccade, gap saccade, and anti-saccade tasks. Using the XGBoost machine learning algorithm, several models were developed based on gait analysis and eye tracking to classify subjective cognitive decline.

**Results:**

A total of 161 gait and eye-tracking features were measured. 22 parameters, including 9 gait and 13 eye-tracking features, showed significant differences between the two groups (*p* < 0.05). The top three eye-tracking paradigms were anti-saccade, gap saccade, and median fixation, with AUCs of 0.911, 0.904, and 0.891, respectively. The gait analysis features had an AUC of 0.862, indicating better discriminatory efficacy compared to the SCD9 scale, which had an AUC of 0.762. The model based on single and dual task gait, anti-saccade, gap saccade, and median fixation achieved the best efficacy in SCD screening (AUC = 0.969).

**Conclusion:**

The gait analysis, eye-tracking multimodal assessment tool is an objective and accurate screening method that showed better detection of subjective cognitive decline. This finding provides another option for early identification of subjective cognitive decline in the community.

## 1 Introduction

In our aging society, the number of people with dementia, particularly Alzheimer’s disease (AD) is increasing year by year (Cao et al., 2020). AD, characterized by progressive cognitive dysfunction and unusual behavior, accounts for 60% of cases (Kalaria et al., 2008), and the number of Chinese AD patients over 60 years old was about 9 million in 2019 (Jia et al., 2020). Patients will gradually lose the ability to take care of themselves, which places a heavy burden on the family and society. The NIA-AA workgroups proposed a model of the clinical trajectory of AD, indicating that once a patient is diagnosed with mild cognitive impairment (MCI), their cognitive function declines more rapidly in the short term compared to the slower progression from the preclinical stage to MCI (Sperling et al., 2011). Therefore, our focus is on early identification and intervention during the preclinical stage. Subjective cognitive decline (SCD) may be considered as a preclinical stage of dementia. Patients with SCD experience memory loss complaints, but their cognitive decline cannot be recognized by standardized neuropsychological scales (Jessen et al., 2014; Lin et al., 2019). The average conversion rate from SCD to cognitive impairment (CI) is 19.8% (Li et al., 2023). Specifically, about 6.6% of older people with SCD will progress to mild cognitive impairment (MCI) annually, while the corresponding number to dementia is approximately 2.3% (Mitchell et al., 2014). Current research is focused on finding objective and simple methods and establishing accurate diagnostic models.

Currently, the diagnosis of SCD relies on a series of assessments, such as neuropsychological scales, imaging, behavioral indicators, and biomarkers. However, there is no uniform standard at present (Jessen et al., 2020). In 2015, a 9-item SCD questionnaire (SCD9) was first developed for SCD screening (Gifford et al., 2015). Researchers validated the value of the Chinese version and found that its sensitivity could reach more than 0.8 (Hao et al., 2017). However, other studies have also indicated that this questionnaire does not encompass all complaints related to cognitive decline (Hao et al., 2022). Therefore, it is necessary to explore a more suitable method for SCD screening for large-scale community surveys in China. Gait and eye tracking are two emerging methods to detect cognitive impairment. Numerous clinical studies assessing MCI and AD through gait and eye movements have produced positive outcomes. The overarching concept is that brain regions and neural circuits related to executive function and attention have been extensively studied and are well recognized. Imaging studies have consistently demonstrated the activity of these brain regions during gait and eye movement tasks. We hypothesize that behavioral changes may emerge at this stage. Furthermore, we aim to identify more accurate, convenient, and validated dual-task gait and eye movement paradigms suitable for large-scale community screening. The specific details are outlined below.

As people age, certain cognitive domains, including processing speed, executive functions, attention, memory, and visuospatial ability tend to decline (Harada et al., 2013). Large-scale studies have shown that over 50% of individuals over 70 years old reported subjective cognitive decline, despite having normal neuropsychological scale results (van Harten et al., 2018). Previous research has confirmed the correlation between gait characteristics and executive function (Ble et al., 2005; Holtzer et al., 2006), with this relationship becoming more pronounced in dual-task gait tests (Coppin et al., 2006). Several hypotheses about attention, a component of executive function, have been used to explain this more evident relationship (Yogev et al., 2008). Recent gait analysis findings related to MCI and AD have also supported the above conclusions (Bahureksa et al., 2017; Bovonsunthonchai et al., 2022). A community survey revealed that slow gait speed may manifest before the beginning of SCD, making it a considerable predictor of future cognitive dysfunction (Merchant et al., 2021). Therefore, it is reliable to study people with SCD in conducting different gait tasks.

Eye tracking involves multiple brain regions, including the cortex, superior colliculus, and thalamus, with different eye-tracking tests engaging distinct brain areas (Tao et al., 2020). Fixation, smooth pursuit, and saccade are the most commonly used methods (Opwonya et al., 2022). Attention and executive control both play crucial roles in eye movement (Luke et al., 2018; Mahon et al., 2018), and eye movement abnormalities may appear before memory deficits (Crawford and Higham, 2016). Recent reports have indicated that eye-tracking tasks can help predict the transition from normal cognition (NC) to cognitive impairment (Zola et al., 2013). A study found that young AD patients performed worse in a fixation task compared to age-matched normal controls (Pavisic et al., 2017). Another meta-analysis suggested that patients with mild cognitive impairment exhibited longer latency, lower accuracy and error correction rate in anti-saccade task (Zhang et al., 2023). Building on a consistent theoretical foundation, we aim to identify differences between individuals with SCD and cognitively normal people through gait and eye-tracking tests. Our goal is to discover more effective tasks for early and efficient identification of the SCD population.

The potential benefits of combining gait analysis and eye-tracking techniques for screening individuals with subjective cognitive decline are still uncertain. Machine learning demonstrates superior performance compared to traditional statistical methods. In our study, we employed the eye-tracking technique for the first time in a community-dwelling population and integrated it with dual-task gait analysis to obtain multimodal parameters for screening individuals with subjective cognitive decline. These parameters were evaluated using a machine learning model. Ultimately, we discovered key parameters for distinguishing the SCD population and developed a high-accuracy diagnostic model for identifying them.

## 2 Materials and methods

### 2.1 Participants

A total of 649 participants aged 65 years or older from three communities in Shanxi Province were selected for this study. Inclusion criteria for gait analysis and eye tracking included: (1) age ≥ 65 years; (2) completion of the cognitive neuropsychological assessments; (3) informed consent signed by the participants themselves or their attendant. Exclusion criteria for gait analysis included: (1) requirement of assistive devices for walking, such as walking sticks; (2) neurological diseases that may affect gait, such as cerebrovascular disease, Parkinson’s disease, ataxia, motor neuron disease, traumatic brain injury; (3) other systemic diseases that may affect gait, such as osteoarthritis, tuberculosis of the spine, limb disability, and audio-visual disorders. Exclusion criteria for eye tracking included: (1) eye diseases that may affect eye tracking tests, such as cataract, glaucoma or other eye diseases; (2) other systematic diseases that may affect vision or eye movement, such as kinetic nerve paralysis; (3) inability to complete the calibration described below before the start of the test.

### 2.2 Neuropsychological assessment

**Figure 1:**
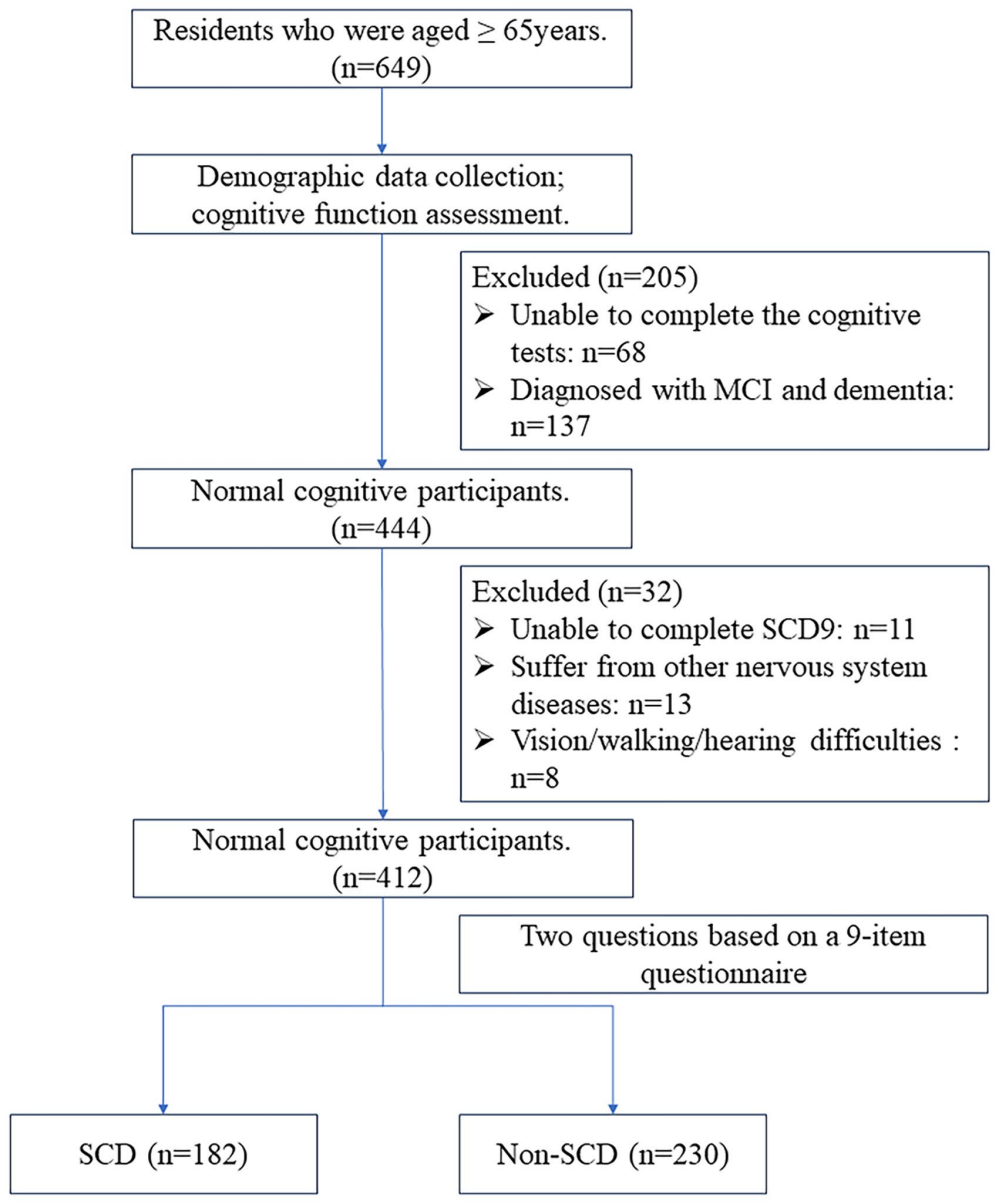
The flow chart for SCD screening. SCD, subjective cognitive decline.

The cognitive evaluators and neurologists in this study received professional training. First, participants scanned the QR code or completed the SCD9 scale in the help of volunteers on the spot. The Ascertain Dementia 8-item Informant Questionnaire (AD8) and Mini-Cog scales were initially assessed by a single assessor. Cognitive function was deemed normal if the AD8 score was <2 and the Mini-Cog score was ≥3. Participants with abnormal scores on either the AD8 or Mini-Cog underwent further assessment. The second stage included the Mini-Mental State Examination (MMSE), Activities of Daily Living (ADL) scale, and Clinical Dementia Rating (CDR). Normal cognition was considered with CDR = 0, while cognitive impairment was CDR ≥ 0.5 (Petersen, 2004). The final diagnosis of CI was determined by two neurologists who took into account the scales, medical history, and educational background to make a comprehensive evaluation. The NC participants were further divided into two groups based on their responses to two questions regarding overall memory function on the SCD9 scale (Cheng et al., 2023). These questions were: “Do you think you have memory problems?” and “Overall, do you tend to forget things you need to do or say?” Responses were either “yes” or “no.” A “yes” to both questions indicated subjective cognitive decline (SCD), while all other responses indicated non-subjective cognitive decline (non-SCD). In total, 412 NC individuals were included: 230 with non-SCD and 182 with SCD. The flow chart of the study is shown in Figure 1. The study was approved by the Ethics Committee of the First Hospital of Shanxi Medical University, and all subjects signed an informed consent form.

### 2.3 Gait analysis

Gait parameters were collected using an artificial motor function assessment system (ReadyGo, Beijing CAS-Ruiyi Information Technology Co., Ltd.). This system incorporates high-accuracy visual sensors and deep learning algorithms to pinpoint 32 skeletal points on the human body. It identifies and labels movements such as foot lifting, arm swinging, and turning, and then calculates the needed gait parameters automatically. The device can capture subjects within a 1 × 5 m^2^ area in front of the camera, so we ensure no other person appears in this space. Subjects do not need to wear sensors, making the process quick and easy. In all tests, subjects were required to walk at their normal speed. For the single-task test, subjects walked along a 3 meter walkway and turned at a designated point for three times, covering a total of 18 meters. For the dual-task test, subjects completed two tasks while walking: (i) counting backwards (e.g., 100, 99, 98, 97, …), (ii) naming as many animals as possible (e.g., monkey, turtle). During the dual-task test, subjects were asked to speak loud enough so that the operator could hear them clearly. The walking process should not be interrupted, even if the next number or animal name was not immediately recalled. We collected gait metrics including stance phase, swing phase, step width, step height, speed, step frequency, stride speed, turnaround time, coordination, step time variation, step width variation, and so on. We also calculated the dual-task cost (DTC) to reflect cognitive load affecting gait performance.

### 2.4 Eye tracking

Eye movement parameters were collected using an intelligent evaluation system (EyeKnow; Beijing CAS-Ruiyi Information Technology Co., Ltd.), with a 120 Hz sampling rate. We used a head-mounted VR glasses and a tablet computer to complete the test and collect data. Before the test, each subject was calibrated using a five-point process: top, bottom, left, right, and center, ensuring a maximum calibration error of 2° radius. The position of the VR glasses remained unchanged after calibration. Subjects performed smooth pursuit, fixation, pro-saccade, and anti-saccade tasks in sequence. Fixation included median fixation and lateral fixation tasks, while pro-saccade included overlap saccade and gap saccade tasks. The specific processes are detailed description as follows in Table 1. If needed, a pretest can be conducted to help subjects understand the process and requirements. In the smooth pursuit task, we analyzed parameters such as tracking accuracy and the number of offsets. In the fixation tasks, we analyzed the offsets, the total offsets, and offsets time. In the pro-saccade tasks, we analyzed accuracy, latency, and speed. In the anti-saccade task, we analyzed accuracy, latency, speed, error correction rate, and error correction time.

**Table 1:** The specific processes of six eye tracking paradigms.

Paradigms	Process	Time
Smooth pursuit	A green circular bright spot first appeared in the center of the screen. The spot will move back and forth in a horizontal line at a speed of 10°/s with a maximum angle of 20°. Participants are required to track the target point in a continuous motion	15 s
Median fixation	A green circular dot appeared in the center of the screen at a 0° viewing angle for 10 s. Participants were asked to maintain their gaze on the target and avoid letting it move from the center of their sight	10 s
Lateral fixation	The system dot appeared at the center for 6 s, then disappeared. Then it reappeared at 15° positions to the left, up, right, and down for 6 s each. Participants were asked to maintain their gaze on the target	30 s
Gap saccade	The system dot appeared at the center for 0.8 s, then disappeared. Then it reappeared for 0.8 s at 20° positions up, down, left, and right at random after a 0.2 s interval. This was for one trail. There were 2 s interval between each trail. Significantly, each test started from the central point and then look around. Subjects were asked to look quickly at the target point when it appeared	This test consisted of 10 trials, totally about 40 s
Overlap saccade	The system dot appeared at the center for 0.8 s, then disappeared. After 0.2 s, the target dot appeared for 0.8 s randomly at 20° positions above and below, left and right. This was for one trail. There were 2 s interval between each trail. Subjects were asked to look quickly at the target point when it appeared	This test consisted of 10 trials, totally about 40 s
Anti-saccade	The system dot appeared at the center for 1 s, then disappeared. A green circular highlight appeared randomly at 20° positions above and below. When the dot appeared at the top, bottom, left, or right, subjects were asked to look quickly in the opposite direction	This test consisted of 10 trials, totally about 40 s

### 2.5 Statistical analysis

**Figure 2:**
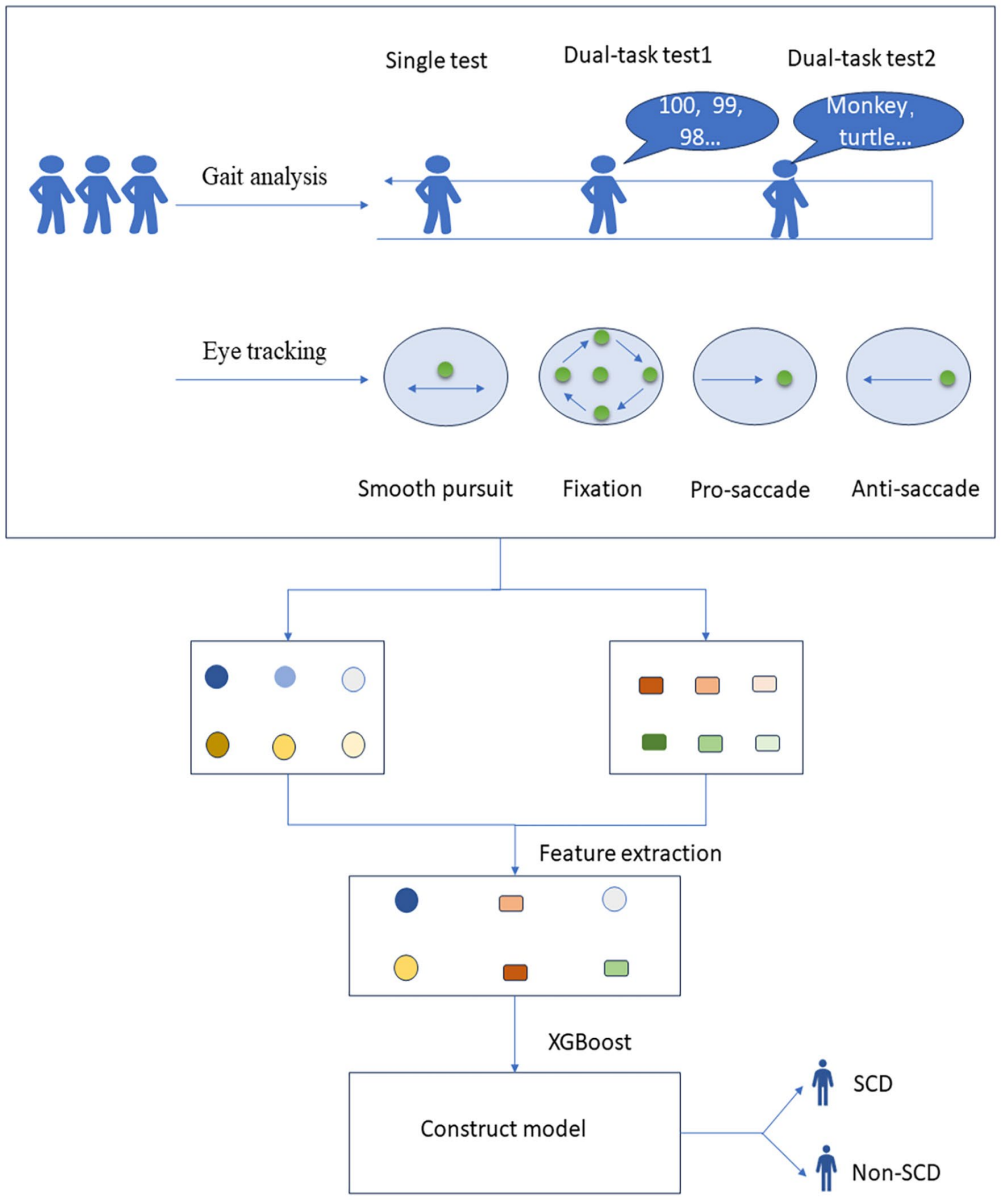
Process of data collection and model construction for gait and eye tracking test. SCD, subjective cognitive decline.

Data were processed using R software version 4.2.3 (Vienna, Austria). Continuous variables were assessed for normality and homogeneity of variance. Data that followed a normal distribution and had uniform variance were expressed as mean ± standard deviation (*x* ± s) and compared using the independent samples *t*-test. Otherwise, they were expressed as the median (interquartile range) [M (QR)] and compared using the Wilcoxon test. Categorical variables were described by frequency and rate (%) and compared using the *χ*^2^ test. A *p*-value <0.05 was considered statistically significant. After excluding outliers and missing values, we used the SHAP (SHapley Additive exPlanation) method for variable selection, a post-hoc model interpretation technique that explains the output of any machine learning model. Then, the dataset was divided into a training set and a test set with a ratio of 7/3. The XGBoost algorithm was used to build a classification model. The model training and evaluation were repeated several times to reduce bias from different dataset divisions. Accuracy, sensitivity, specificity, and area under the curve (AUC) were used as comprehensive measures for model evaluation. Model stability was assessed using a 5-fold cross-validation method. In each iteration, four subsets are selected as the training set, and the remaining subset is used as the test set. The training set is used to train the model, and the test set is used to evaluate the performance of the model, and evaluation metrics such as accuracy, sensitivity, specificity, and AUC are calculated. The evaluation results on the test set in all iterations are summarized and average metrics are calculated. A total of 12 models were constructed (Single-task gait (ST), Dual-task gait (counting backward, CB), Dual-task gait (naming animals, NA), Single-task gait + Dual-task gait (ST + DT), Smooth pursuit (SP), Median fixation (MF), Lateral fixation (LF), Overlap saccade (OS), Gap saccade (GS), Anti-saccade (AS), Anti-saccade + Median fixation + Gap saccade (AS + MF + GS), Single-task gait + Dual-task gait + Anti-saccade + Median fixation + Gap saccade (ST + DT + AS + MF + GS)), and different modality combinations were compared. The details and results are presented in Table 2. The data collection and model-building process are shown in Figure 2.

**Table 2:** Area under ROC curve and statistics of different models of two groups.

Feature	AUC	Accuracy (%)	Sensitivity (%)	Specificity (%)
SCD9	0.762	75.30	72.53	78.36
Single-task gait (ST)	0.823	83.64	86.31	82.64
Dual-task gait (counting backward, CB)	0.824	85.34	86.98	82.89
Dual-task gait (naming animals, NA)	0.841	85.98	87.32	83.37
Single-task gait + Dual-task gait (ST + DT)	0.862	86.43	87.78	85.61
Smooth pursuit (SP)	0.842	83.05	89.13	83.12
Median fixation (MF)	0.891	85.34	89.03	86.23
Lateral fixation (LF)	0.873	84.31	86.89	86.01
Overlap saccade (OS)	0.866	85.03	85.97	84.43
Gap saccade (GS)	0.904	88.18	87.03	91.21
Anti-saccade (AS)	0.911	92.01	89.26	92.86
Anti-saccade + Median fixation + Gap saccade (AS + MF + GS)	0.919	92.44	90.34	93.01
Single-task gait + Dual-task gait + Anti-saccade + Median fixation + Gap saccade (ST + DT + AS + MF + GS)	0.969	94.82	96.72	95.21

## 3 Results

### 3.1 Demographic and clinical characteristics

We recruited 649 participants from three communities in Shanxi Province for this study. Among them, 412 were diagnosed with normal cognition (NC): 230 with non-subjective cognitive decline (non-SCD) and 182 with subjective cognitive decline (SCD). Table 3 summarizes the baseline characteristics of the participants. Age, years of education, and BMI between the two groups showed no statistically significant differences (*p* > 0.05). There were more females in the SCD group compared to the non-SCD group, with a statistically significant difference (*p* < 0.05). The SCD9 score was significantly higher in the SCD group than in the non-SCD, with a statistically significant difference (*p* < 0.05).

**Table 3:** Basic information comparisons of two groups.

Demographics	Non-SCD (*N* = 230)	SCD (*N* = 182)	*p*-value
Age, years	70.67 ± 5.06	71.01 ± 5.73	0.2646
Females, *n* (%)	124 (53.9)	123 (67.6)	<0.001
Education, years	9.14 ± 4.03	9.76 ± 3.60	0.1394
BMI	23.17 ± 5.76	23.45 ± 4.71	0.3925
SCD9 score	2.84 ± 1.79	6.01 ± 1.49	<0.001

BMI, body mass index; SCD9, a 9-item questionnaire for SCD screening; SCD, subjective cognitive decline.

### 3.2 Gait parameters and eye tracking parameters with differences between non-SCD and SCD groups

**Figure 3:**
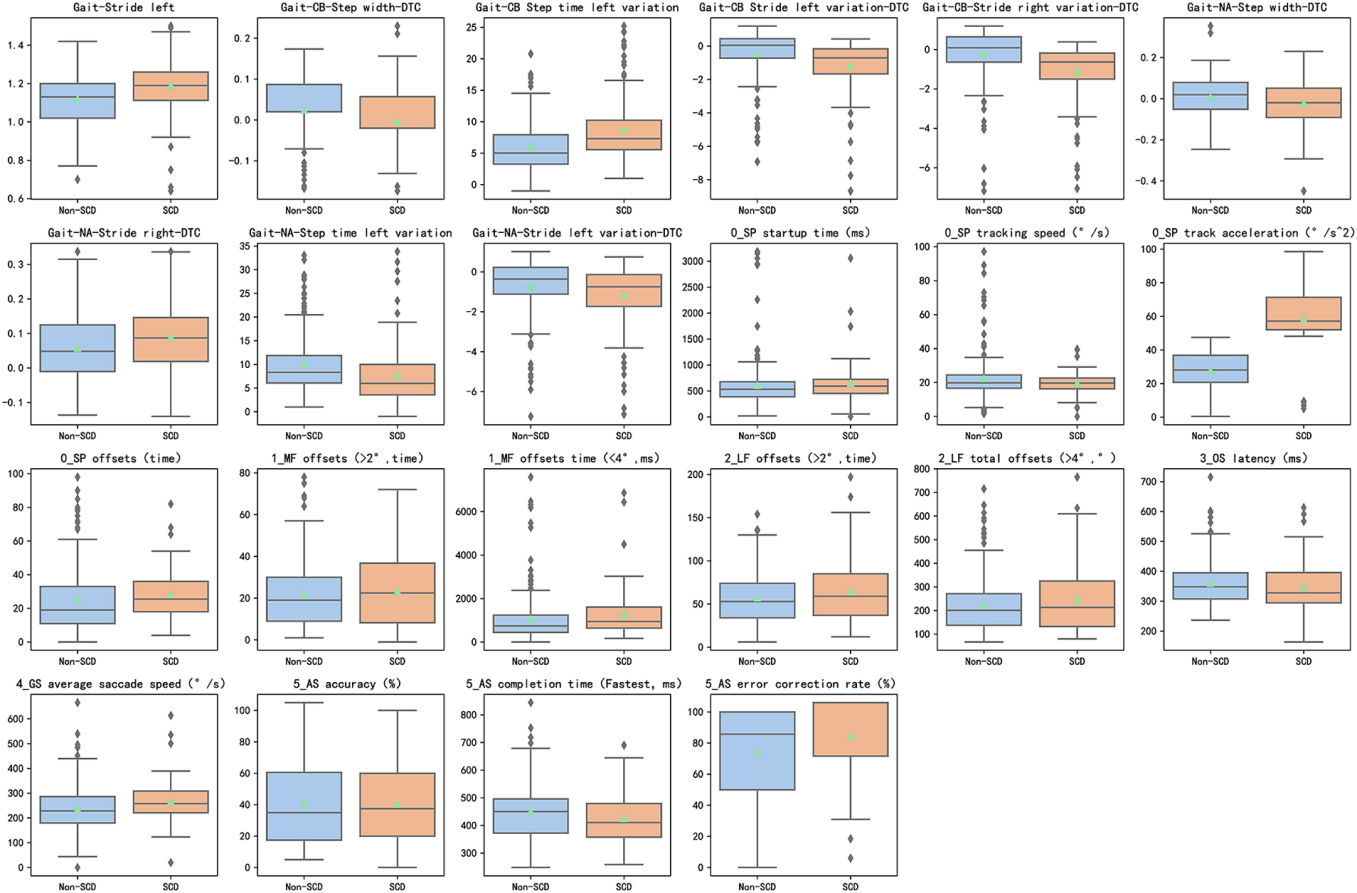
9 distinctive gait features and 13 distinctive eye tracking features (*p* < 0.05) between the SCD and non-SCD groups. SCD, subjective cognitive decline; DTC (dual-task cost) = ([single-task parameter − dual-task parameter]/single-task parameter) × 100%.

We collected a total of 125 gait parameters and 36 eye-tracking parameters. Then, 9 gait parameters and 13 eye tracking parameters were found significantly different between the two groups, as shown in Figure 3. In the single-task test, only the parameter of stride left was found significant difference between the two groups. About dual-task tests, three parameters including step width-DTC, step time left variation, and stride left variation-DTC, had statistical significance in two types of tests between two groups. In eye tracking paradigms, offsets were significantly different in smooth pursuit, median fixation, and lateral fixation between non-SCD and SCD Groups. In the anti-saccade test, accuracy, completion time, and error correction rate showed statistically significant differences in the two groups.

### 3.3 Performance of gait models for SCD detection

**Figure 4:**
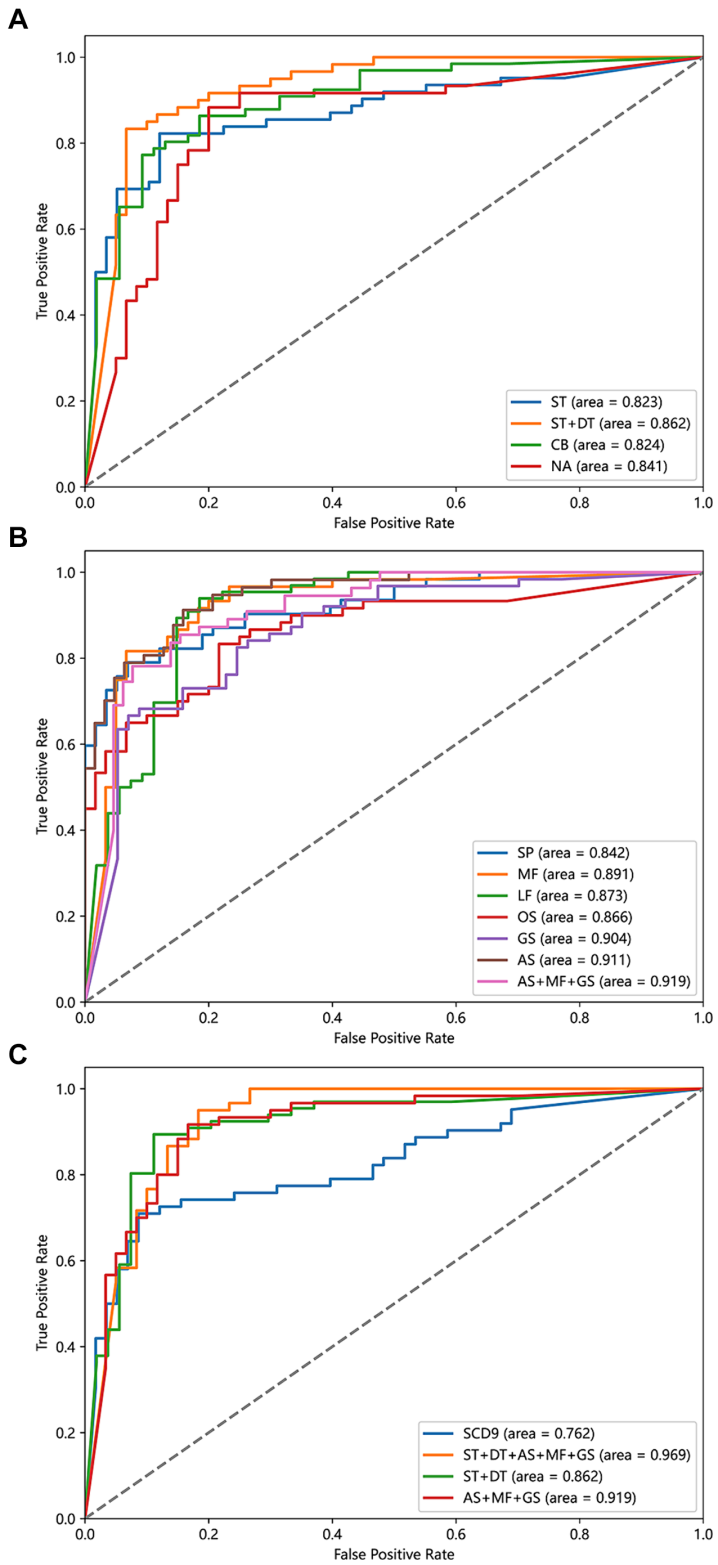
Power of different detect models to discriminate SCD and non-SCD population. **(A)** Models based on ST, CB, NA, and ST + DT features. **(B)** Models based on SP, MF, LF, OS, GS, AS, and AS + MF + GS features. **(C)** Models based on SCD9, ST + DT, AS + MF + GS, ST + DT + AS + MF + GS features. ST, the single task gait; DT, the dual task gait; CB, the counting backwards dual-task gait; NA, the naming animals dual-task gait; SP, smooth pursuit; MF, median fixation; LF, lateral fixation; OS, overlap saccade; GS, gap saccade; AS, anti-saccade.

To evaluate the effectiveness of single-task and dual-task gait tests for SCD detection, we generated four machine learning models and evaluated the discriminative abilities of the single-task test, the counting backwards dual-task test, the naming animals dual-task test, and the combination of single-task and two dual-task tests, as shown in Figure 4A. We aimed to determine whether the dual-tasks were superior to the single-task and to compare the two dual-tasks in order to identify the more effective task for further research. Additionally, we sought to understand the extent to which combining a single task with a dual-task enhances discriminative ability. The model based on naming animals dual-task gait traits (AUC: 0.841) discriminated between SCD and non-SCD populations better than the model based on single-task gait traits (AUC: 0.823), which had a similar discriminatory ability as the counting backwards dual-task gait model (AUC: 0.824). The model combining single-task and two dual-task gait features (AUC: 0.862) outperformed all other gait models.

### 3.4 Performance of eye tracking models in SCD detection

To evaluate the effectiveness of different eye-tracking paradigms for SCD detection, we generated seven machine learning models and evaluated them using metrics such as AUC, accuracy, sensitivity, and specificity. Previously, no consensus existed on which eye-tracking task was superior, and the specific processes used varied between studies. Therefore, we sought to determine which eye-tracking paradigm is superior and whether combined features can improve the results. We first evaluated the discrimination abilities of the smooth pursuit, median fixation, lateral fixation, overlap saccade, gap saccade, and anti-saccade tasks. Among the six eye-tracking paradigms, the classification model using parameters in the anti-saccade task had the highest accuracy (AUC: 0.911), while the next two better models were gap saccade (AUC: 0.904) and median fixation (AUC: 0.891). In addition, the smooth pursuit paradigm had the lowest accuracy (AUC: 0.842). Then, we combined the three paradigms with the highest AUCs to build a comprehensive model and found that the classification model using the anti-saccade, gap saccade, and median fixation tasks had the highest ability (AUC: 0.919), surpassing any single eye-tracking paradigm model to discriminate the SCD and non-SCD people, as shown in Figure 4B.

### 3.5 Performance of the multimodal for SCD detection

We attempted to compare the diagnostic value of the traditional scale with emerging behavioral assessment tools, such as gait analysis and eye tracking, in distinguishing SCD populations. Therefore, we compared four models: SCD9, gait single-task + dual-task, anti-saccade + gap saccade + median fixation, and gait single-task + dual-task + anti-saccade + gap saccade + median fixation. The results are shown in Figure 4C. Among the four classification models, the gait and eye tracking combination model was the most effective, with an AUC of 0.969, superior to single gait or eye tracking models. The SCD9 model had the lowest classification ability (AUC: 0.762).

### 3.6 Validation of gender difference and other machine learning models

As shown in Table 3, there was a significant difference in the gender factor between the two groups. To assess the impact of gender differences, we recalculated the diagnostic model by including gender as a factor, with the results displayed in Table 4. From these results, there was no significant change in the performance of model after adjusting for gender. Additionally, we compared four commonly used machine learning models, such as logistic regression, RF, SVM, and LightGBM, in addition to XGBoost to explore their detection abilities. The results indicated that XGBoost performed best, followed by LightGBM. Specific details are shown in Table 5.

**Table 4:** Area under ROC curve and statistics of different models of two groups adjusted for gender.

Feature	AUC	Accuracy (%)	Sensitivity (%)	Specificity (%)
SCD9	0.762	75.30	72.53	78.36
Single-task gait (ST)	0.826	83.31	86.19	82.83
Dual-task gait (counting backward, CB)	0.823	85.27	87.01	82.93
Dual-task gait (naming animals, NA)	0.841	85.95	87.45	83.34
Single-task gait + Dual-task gait (ST + DT)	0.861	86.23	87.78	85.61
Smooth pursuit (SP)	0.843	83.08	89.23	83.10
Median fixation (MF)	0.893	85.34	89.04	86.27
Lateral fixation (LF)	0.871	84.43	86.60	86.05
Overlap saccade (OS)	0.865	85.03	85.97	84.34
Gap saccade (GS)	0.903	88.18	87.12	91.25
Anti-saccade (AS)	0.910	92.05	89.34	92.89
Anti-saccade + Median fixation + Gap saccade (AS + MF + GS)	0.920	92.65	90.45	93.04
Single-task gait + Dual-task gait + Anti-saccade + Median fixation + Gap saccade (ST + DT + AS + MF + GS)	0.963	94.67	95.99	95.34

**Table 5:** Main results of different machine learning models.

Feature	Machine learning	AUC	Accuracy (%)	Sensitivity (%)	Specificity (%)
Single-task gait + Dual-task gait (ST + DT)	Logistic regression	0.725	74.25	76.26	75.37
	RF	0.812	84.26	82.64	80.55
	SVM	0.793	80.64	81.35	81.54
	LightGBM	0.853	84.36	85.37	82.74
	XGBoost	0.862	86.43	87.78	85.61
Anti-saccade + Median fixation + Gap saccade (AS + MF + GS)	Logistic regression	0.742	79.25	78.43	80.74
	RF	0.842	85.31	83.84	81.42
	SVM	0.812	82.52	83.53	82.69
	LightGBM	0.904	93.12	89.34	92.42
	XGBoost	0.919	92.44	90.34	93.01
Single-task gait + Dual-task gait + Anti-saccade + Median fixation + Gap saccade (ST + DT + AS + MF + GS)	Logistic regression	0.783	82.31	84.21	81.53
	RF	0.857	87.32	86.12	83.21
	SVM	0.832	84.24	85.15	83.94
	LightGBM	0.954	94.23	95.12	94.74
	XGBoost	0.969	94.82	96.72	95.21

## 4 Discussion

This study used eye-tracking technology for the initial screening of SCD and combined it with gait single-task and dual-task tests, expecting to explore a new and effective screening method for SCD. Nine gait parameters and thirteen eye-tracking parameters were found significantly different between non-SCD and SCD individuals. Using the machine learning classification models constructed in this study, we found that combining gait and eye tracking achieved the best result (AUC: 0.969) in distinguishing between non-SCD and SCD individuals. This outperformed the traditional SCD9 scale (AUC: 0.762) in terms of diagnostic accuracy. In previous studies, similar positive results have been observed in neurodegeneration diseases using these methods. One study investigated cognitive impairment using gait, speech, and drawing, achieving a combined model with an accuracy was 0.93 (Yamada et al., 2021). Additionally, another large-scale community study using dual-task gait and eye movement achieved an AUC of 0.987 for cognitive impairment detection (Lin et al., 2023). Both studies employed machining learning algorithms. Thus, we believe that this screening tool is valuable for identifying SCD in community-dwelling elderly population in the future.

Currently, research on gait analysis involves various devices to collect gait parameters and dual-task paradigms to identify cognitive decline. The equipment used in this study integrates video recording, parameter acquisition and analysis, with no need of additional sensors or video recording equipment, which makes it a more practical tool, suitable for the large-scale community screening. In this study, two types of dual-task tests were used, including counting backwards and naming animals, which have been found to be efficient in discriminating subjective cognitive impairment (Åhman et al., 2020). The ability of counting backwards test modal (AUC: 0.824) to detect SCD was not found to be significantly better than the single test gait (AUC: 0.823), suggesting its limitation in distinguishing SCD. The theory behind dual-task tests assumes that attentional resources are limited, and performance declines when two attention-demanding are performed simultaneously (Tombu and Jolicoeur, 2003). Therefore, it may be relatively simple and less cognitively demanding for the SCD population to conducted counting task (Montero-Odasso et al., 2017), consistent with previous findings (Tseng et al., 2014), similar to the task of recitation of the alphabet (Ghoraani et al., 2021). When analyzing data from the naming animals dual-task model, we found that it can enhance the ability of the single task and counting backwards dual task to discriminate SCD individuals with an AUC of 0.841. A cross-sectional study also found that the knee peak extension angle in naming animals dual-task test differed in distinguishing different cognitive groups (Ali et al., 2022). The animal-naming test assesses verbal fluency, requiring extensive knowledge and active retrieval processes. In a comparison study of three dual-task tests, researchers found that the tasks of subsequent 100-7 and naming animals had similar effects on gait performance in the MCI group (Du et al., 2023). For SCD subjects, the animal-naming task posed a higher cognitive demand than counting backward. Some gait characteristics were observed to be different in the two groups such as stride, step width and step time variation. This may be attributed to the shared neural pathways between gait and cognition, particularly in the prefrontal cortex, temporal regions, and entorhinal cortex (Sakurai et al., 2019), which are connected with executive function. Disruption caused by neurodegenerative processes may impact both functions (Grande et al., 2019). Future studies should further explore other valuable dual tasks for differentiating SCD.

Previous studies have shown that eye-tracking techniques are valuable in distinguishing between cognitively impaired and cognitively normal populations. Oyama et al. (2019) found that eye-tracking had good diagnostic performance in detecting patients with cognitive impairment (AUC: 0.888). In a study using pro-saccade and anti-saccade task, saccade behavior exhibited more errors, omissions, and fewer corrections in the MCI group compared to cognitively normal individuals, suggesting the probability of saccade tasks as cognitive markers of MCI (Chehrehnegar et al., 2022). Pro-saccade and anti-saccade involve activation of frontal and parietal lobes, lenticular nuclei, and occipital cortex which are responsible for planning and executing saccadic eye movement (Matsuda et al., 2004). Few of eye-tracking research studies have focused on the SCD population, especially in community survey. Xue et al. (2022) adopted a visual search task and found that gaze duration and times in the area of interest were increased in SCD patients, potentially enabling differentiation between SCD and NC. Taken together, this suggests the potential for recognizing SCD using eye-tracking technologies. In our study, we found significant differences of offsets and offset time in the median fixation task and offsets and total offsets in the lateral fixation task. One possible reason for the different results of the two studies is that we used different tasks and stimulators. To better understand the paradigms of eye tracking in SCD people, particularly at the community level, we utilized six tasks including smooth pursuit, median fixation, lateral fixation, overlap saccade, gap saccade, and anti-saccade tasks to advance our research. The anti-saccade (AUC: 0.911), gap saccade (AUC: 0.904), and median fixation (AUC: 0.891) paradigms had the strongest differentiation ability. Similar to our results, prior studies on cognitive impairment also proved the value of these three tasks (Wolf and Ueda, 2021; Lin et al., 2023; Wolf et al., 2023). We initially constructed a combination model of anti-saccade, gap saccade, median fixation and found better results (AUC: 0.919). The theory of detecting cognitive decline using eye-tracking parameters is still unknown. Researchers found that eye-tracking parameters have a correlation with neuropsychological scale scores. Total eye-tracking scores significantly decreased in CI population, correlating favorably with the scores on the MMSE (Tadokoro et al., 2021). In addition, eye tracking tests show an advantage over scales in that they have less impact on educational attainment. Also, fixation, saccade, and smooth pursuit, as demonstrated in prior studies, require the engagement of various cortical and subcortical regions including the frontal cortex and anterior cingulate cortex, reflecting the executive function and attention. Hence, eye-tracking assessments offer promise in cognitive function evaluation (Tao et al., 2020). To further explore whether combining gait and eye movement enhances SCD recognition and to investigate a model with superior classification, we fused data from the three eye tracking paradigms with the best classification performance with gait single-task and dual-task paradigms. This integration yielded the most successful model with an AUC of 0.969, suggesting the value of multimodal analysis. Remarkably, this study was the first to integrate eye-tracking tasks with a machine learning algorithm for community-based SCD screening. Future investigations should delve into paradigms which can offer enhanced classification efficacy and develop more intelligent evaluation models using eye-tracking technologies.

This study combined eye tracking and gait data, yielding improved outcomes. While the accuracy of these behavioral parameter models may not be equal to cerebrospinal fluid biomarkers and imaging like PET-CT, they surpass the traditional SCD9 scale in advantage of accuracy, objectivity and simplicity. These models are anticipated to emerge as validated tools for distinguishing SCD, MCI, and dementia in the future. In conclusion, our study demonstrates, for the first time, the effectiveness of combining eye tracking and dual-task gait analysis for SCD assessment, leading to promising results. This innovative approach shows significant potential for widespread clinical and community screening in the future.

Our study possesses several limitations. Firstly, the sample size was only 412 elderly individuals aged over 65 from three communities, which may limit the generalizability of the findings. Future research should aim to validate the conclusions in larger cohorts. Secondly, the average education level of approximately 9 years may not accurately represent individuals with lower education levels, such as those who are illiterate or have only completed primary education, thus restricting the generalizability of our findings to those with lower education. Future studies should validate these results across diverse populations with varying living conditions and cultural backgrounds. Lastly, our study adopted a cross-sectional design, which means we could not observe the progression of SCD and its association with MCI and dementia. Future endeavors should include longitudinal follow-up to understand how multimodal behavioral parameters evolve in SCD progression.

## 5 Conclusion

Overall, we observed that the naming animals dual task showed better discriminatory value in SCD detection compared to the counting backwards dual task, thereby enhancing the diagnostic capability of single-task gait analysis. Among the eye-tracking tasks, the anti-saccade, gap saccade, and median fixation paradigms exhibited the highest classification efficacy. Combining these three paradigms with gait analysis yielded an optimal classification model. These findings imply that the integration of eye-tracking and gait analysis has the potential to improve early SCD diagnosis, support the development of early intervention strategies and brain health management, and could be useful for widespread use in community screening programs.

## Data Availability

The original contributions presented in the study are included in the article/supplementary material, further inquiries can be directed to the corresponding author.
